# Overexpression of the *β-Glucosidase* Gene *SpBGLU25* from the Desert Pioneer Plant *Stipagrostis pennata* Enhances the Drought Tolerance in *Arabidopsis*

**DOI:** 10.3390/ijms26146663

**Published:** 2025-07-11

**Authors:** Jiahuan Niu, Jingru Wang, Faren Zhu, Xuechi Li, Jianting Feng, Jiliang Fan, Mingsu Chen, Xiaoying Li, Ming Hu, Zhangqi Song, Zihan Li, Fei Wang, Rong Li, Hongbin Li

**Affiliations:** 1 Key Laboratory of Xinjiang Phytomedicine Resource and Utilization of Ministry of Education, Key Laboratory of Oasis Town and Mountain-Basin System Ecology of Bingtuan, College of Life Sciences, Shihezi University, Shihezi 832000, China; 17599931126@163.com (J.N.); 18935768685@163.com (J.W.); zhufaren163@163.com (F.Z.); fanplanbeson@163.com (J.F.);; 2 Department of Civil, Environmental, and Construction Engineering, College of Engineering and Computer Science, University of Central Florida, Orlando, FL 32816, USA

**Keywords:** *Stipagrostis pennata*, *SpBGLU25*, drought stress tolerance, bioinformatics analysis, expression analysis, plant hormones

## Abstract

This research centers on the sand-fixing plant known as *Stipagrostis pennata*, from which the β-glucosidase gene *SpBGLU25* was successfully cloned using the molecular cloning method. *SpBGLU25* encodes a hydrophilic and stable protein made up of 193 amino acids, located in the cell membrane. qRT-PCR analysis indicated that the expression of the *SpBGLU25* is closely linked to the drought stress tolerance of *S. pennata*. Following this, functional validation was performed using an *Arabidopsis* overexpression system. The overexpression of transgenic *Arabidopsis* lines showed significantly improved drought tolerance under PEG and mannitol treatments. Assessments of germination, root length, and physiological indicators such as proline, malondialdehyde content, soluble sugars, and relative leaf water content (RLWC) further confirmed the enhanced performance of the overexpressing plants. Additionally, the comparative transcriptomic analysis of *SpBGLU25-OE Arabidopsis* compared to the wild-type (WT) showed that differentially upregulated genes were primarily enriched in categories of “cellular process,” “cell,” and “catalytic activity.” KEGG pathway enrichment analysis indicated that the genes were mainly concentrated in the pathways of phenylpropanoid biosynthesis and plant hormone signal transduction. These findings provide a crucial foundation for further investigation into the function of the *SpBGLU25* and its role in regulating plant tissue development and adaptation to stress. This research is anticipated to offer new theoretical insights and genetic resources for enhancing plant stress tolerance through genetic engineering.

## 1. Introduction

*Stipagrostis pennata* is a pioneering plant species found in the deserts of Xinjiang, China, where it plays a crucial role in stabilizing sand dunes and is commonly associated with sandy environments [1,2,3]. This plant thrives under conditions characterized by persistent dryness, low rainfall, significant wind erosion, and a high degree of soil sandyization [4,5,6]. Consequently, *S. pennata* has developed several adaptations for survival in desert ecosystems, including drought resistance, tolerance to wind erosion, and the ability to withstand sand burial [7]. Its unique rhizosheath structure allows it to flourish in extreme conditions, such as drought and high temperatures, while also enhancing sand dune stability and promoting plant diversity, highlighting its potential for ecological restoration [3,8]. Therefore, studying the drought-resistant *S. pennata* in this region holds considerable scientific and practical significance. Plants respond to abiotic stress through complex physiological, biochemical reactions, and molecular regulatory mechanisms [9], with enzymatic substances playing a crucial role in these processes. β-glucosidase, an important hydrolase, is significantly involved in various physiological functions, including plant growth and development, secondary metabolite synthesis, and stress responses [10,11]. As a member of the glycoside hydrolase family I, β-glucosidase catalyzes the hydrolysis of β-glycosidic bonds, thus participating in plant metabolism to regulate growth and development [12,13]. Based on amino acid sequences and conserved domain similarities, plant β-glucosidases can be categorized into eight families: GH1, GH3, GH5, GH7, GH9, GH12, GH35, and GH116 [14,15]. In plants, β-glucosidases play roles in various physiological processes, including stress defense responses, hormone metabolism, cell wall lignification, and carbohydrate metabolism [13,16]. β-glucosidase plays a crucial role in cell wall metabolism by catalyzing the hydrolysis of polysaccharides, which impacts the structure and function of the cell wall. This, in turn, affects plant cell growth, differentiation, and overall morphological development [17,18]. Lignin, a key component of the cell wall, is maintained by β-glucosidase through the degradation of oligosaccharides and the release of lignin monomers from glycosides [19,20,21]. In response to abiotic stressors like drought, high temperatures, and salt, β-glucosidase enhances plant tolerance by regulating internal signaling pathways and participating in the synthesis and metabolism of plant hormones [22]. Recent research has shown that the β-glucosidase *AtBG1* can hydrolyze ABA-GE (ABA-glucosyl ester) to produce active free ABA, which is crucial for the plant’s response to dehydration [23,24,25]. The β-glucosidase gene is involved in polysaccharide metabolism, as indicated by metabolomics studies, and plays a role in the development and stress resistance of various plant organs, including rice leaves and seeds [26]. Additionally, β-glucosidase is linked to the EMP glycolytic pathway and is vital for sugar metabolism, supporting normal physiological functions [14,17].

Despite the growing recognition of β-glucosidase’s importance in plants, research on the β-glucosidase gene in desert plants like *S. pennata* is limited. Conducting in-depth studies on the structure, function, and regulatory mechanisms of the β-glucosidase gene in *S. pennata* under abiotic stress will help uncover how this plant adapts to desert conditions. This research could also provide valuable genetic resources and theoretical insights for using genetic engineering to enhance stress resistance in other plants, which is essential for advancing desert ecological restoration, agricultural production, and plant stress resistance research. Therefore, this study focuses on cloning and analyzing the expression of the β-glucosidase gene *SpBGLU25* in *S. pennata*, aiming to establish a foundation for a better understanding of its stress resistance mechanisms.

## 2. Results

### 2.1. Phylogenetic Analysis of the SpBGLU25 Gene Sequence in Different Closely Related Species

**Figure 1:**
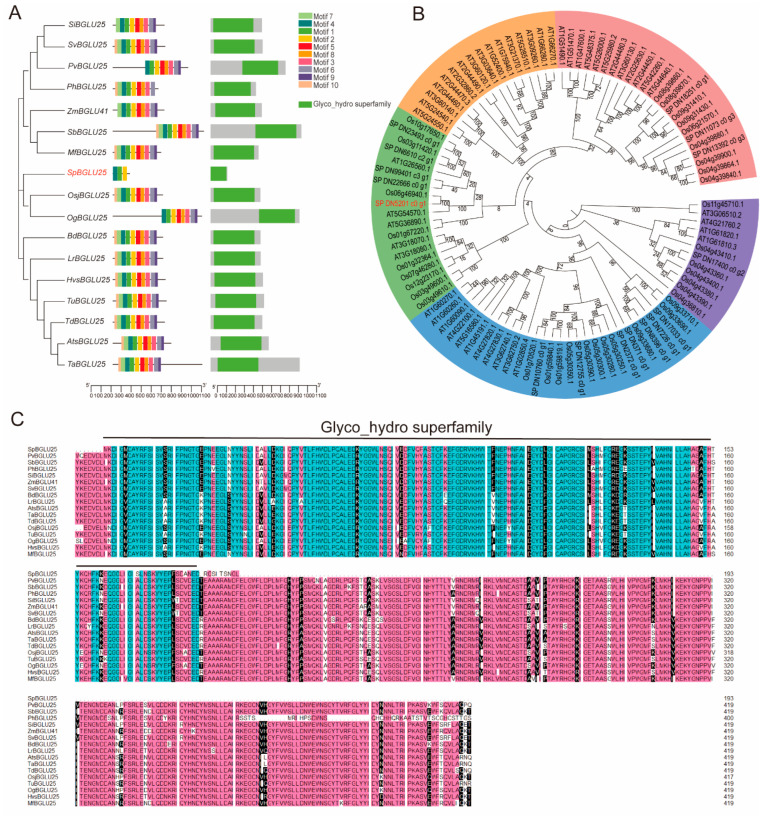
Multiple sequence alignment and motif analysis of BGLU from different species. (**A**): Structural domain and motif analysis of BGLU from different species. (**B**): Phylogenetic analysis of the BGLU family in *Arabidopsis*, rice, and *S. pennata* species. (**C**): Multiple sequence alignment of BGLU from different species.

A BLAST comparison of the *SpBGLU25* protein was conducted using the NCBI database, resulting in the selection of the top 32 homologous genes with the highest sequence similarity for further analysis. The findings revealed that these genes are predominantly found in 17 species of monocots, with one sequence from each species exhibiting the greatest homology to the BGLU gene. Phylogenetic and sequence alignment analyses were performed using MEGA and DNAman, respectively, demonstrating that these genes share certain similarities. Domain analysis indicated that all BGLUs possess a common conserved domain from the Glyco_hydro superfamily (Figure 1A,C). While *SpBGLU* contains three conserved motifs, all other genes have more than five motifs, underscoring the high conservation of BGLU (Figure 1A).

To further investigate the evolutionary relationships of the BGLU genes, we identified members of the BGLU gene family in rice and *Arabidopsis* and constructed a phylogenetic tree for BGLU family members from *S. pennata*, rice, and *Arabidopsis*, as illustrated in the figure. The phylogenetic tree categorizes BGLU gene members from the three species into five subfamilies. Among these species, the BGLU members in rice are most closely related to those in *S. pennata*, while the BGLU members in *Arabidopsis* are more distantly related to those in *S. pennata* (Figure 1B).

### 2.2. Expression Analysis of SpBGLU25

**Figure 2:**
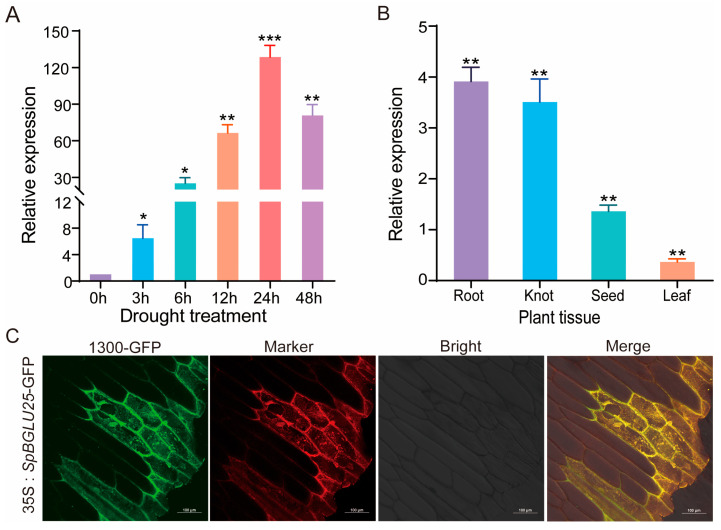
Expression analysis of the *SpBGLU25* gene (**A**): Expression levels of the *SpBGLU25* gene under different drought stress durations. (**B**): Expression of the *SpBGLU25* gene in different tissues of *S. pennata*. (**C**): Subcellular localization of the *SpBGLU25* protein in onion. (Green is the 1300-GFP fluorescent protein, red is the cell membrane marker, and yellow is due to the overlap of green and red fluorescence, indicating the result of colocalization). Note: *, **, and *** denoting significant differences at *p* < 0.05, 0.01, and 0.001 levels, respectively.

To further investigate the expression changes in the β-glucosidase gene in various tissues of *S. pennata* under drought stress, samples were collected from the roots, nodes, leaves, and seeds. These samples were obtained at intervals of 0 h, 3 h, 6 h, 12 h, and 24 h following drought treatment and were analyzed using RT-qPCR. The quantitative PCR results indicated a significant increase in the expression of the *SpBGLU25* gene after exposure to drought stress (Figure 2A). Analysis of tissue specificity revealed that the *SpBGLU25* gene was predominantly expressed in the roots, nodes, and seeds, with the highest levels found in the root tissue (Figure 2B). These findings strongly suggest that the *SpBGLU25* gene responds positively to drought stress. Additionally, a *35S::SpBGLU25-GFP* vector was constructed, and the recombinant plasmid was introduced into onion cells. Subcellular localization was examined using a laser confocal microscope, which demonstrated that *SpBGLU25* is expressed in the cell membrane of the onion cells (Figure 2C), aligning perfectly with earlier predictions.

### 2.3. SpBGLU25 Involvement in Plant Response to Drought Stress

#### 2.3.1. *SpBGLU25* Enhances Seed Germination Vitality and Root Growth of *Arabidopsis* Under Drought Stress

**Figure 3:**
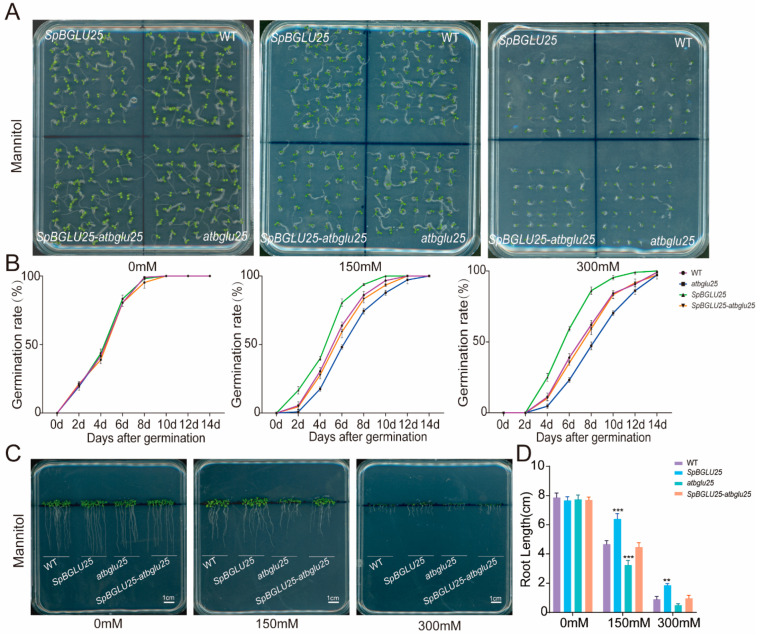
Germination rate and root length detection of *Arabidopsis* seeds overexpressing *SpBGLU25* under drought stress. (**A**): Phenotypic images of germination rates of four *Arabidopsis* types under different concentrations of mannitol simulating drought stress. (**B**): Statistical chart of germination rates of four *Arabidopsis* types under different concentrations of mannitol simulating drought stress. (**C**): Phenotypic images of root lengths of four *Arabidopsis* types under different concentrations of mannitol simulating drought stress. (**D**): Statistical chart of root lengths of four *Arabidopsis* types under different concentrations of mannitol simulating drought stress. Note: ** and *** denoting significant differences at *p* < 0.01 and 0.001 levels, respectively.

To evaluate the drought stress tolerance of *SpBGLU25* transgenic *Arabidopsis* under sterile conditions, this study involved inoculating seeds from four *Arabidopsis* types: wild type (WT), *SpBGLU25-OE*, *atbglu25* knockout, and *SpBGLU25-atbglu25* complementation, onto 1/2 MS medium with varying concentrations of (0 mM, 150 mM, and 300 mM) to assess seed germination rates and root growth. The results indicated no significant differences in germination rates among the four types on the 1/2 MS medium without mannitol. However, under drought stress conditions induced by mannitol, the germination rate of the *SpBGLU25* transgenic types was significantly higher than that of the wild type (Figure 3A). Specifically, the germination rates for the *SpBGLU25* transgenic plants approached nearly 100% by the 8th and 10th days, while the *atbglu25* knockout mutant seeds only reached similar rates by the 12th and 14th days. A line graph illustrating the germination rate over time showed that the *SpBGLU25* transgenic *Arabidopsis* had the highest germination rate among the four types (Figure 3B). As mannitol concentration increased, the root lengths for all four types were inhibited (Figure 3C). Under 150 mM and 300 mM mannitol treatments, the *SpBGLU25* transgenic type exhibited less root length inhibition compared to the wild type, with root elongations of 6.4 cm and 1.87 cm, respectively, compared to 4.7 cm and 0.9 cm for the wild type (Figure 3D). The root system serves as the primary organ for water uptake in plants. *Arabidopsis* increases its root length to expand its water absorption capacity, a vital adaptation for plants experiencing drought conditions. These findings suggest that the *SpBGLU25* gene may enhance drought stress tolerance in *Arabidopsis*.

#### 2.3.2. *SpBGLU25* Significantly Enhances Drought Resistance in *Arabidopsis* by Strengthening Antioxidant Defense and Osmotic Regulation

**Figure 4:**
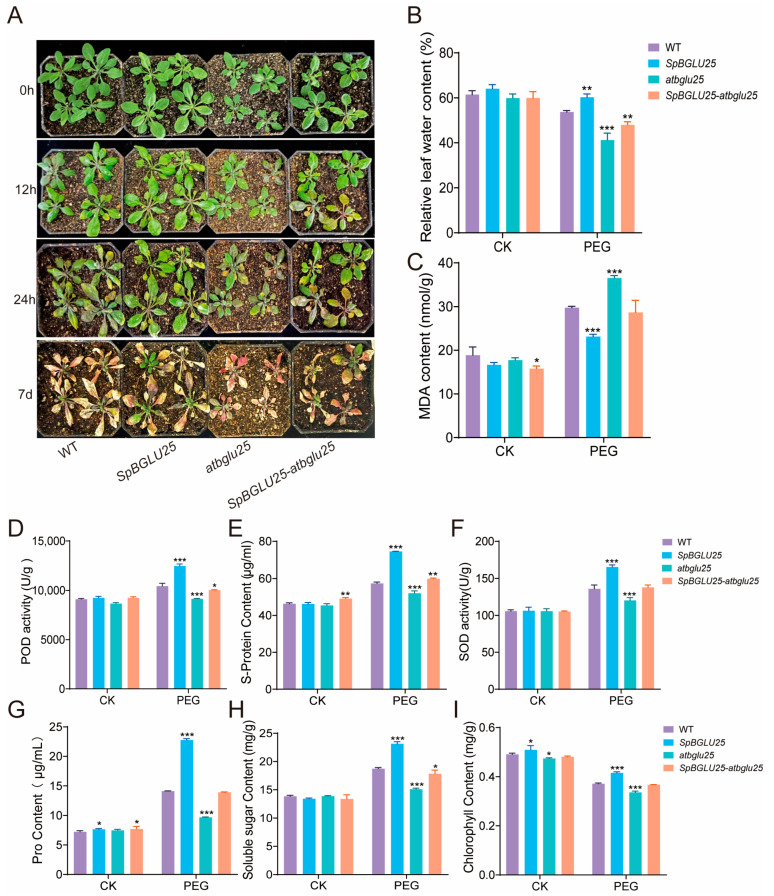
Effects of drought stress on the phenotype of *SpBGLU25* gene overexpressing *Arabidopsis* and its physiological indicators measurement. (**A**): Phenotype images of four types of *Arabidopsis* under drought stress. (**B**): RLWC measurement of four types of *Arabidopsis* under drought stress. (**C**): MDA content. (**D**): POD activity. (**E**): Soluble protein content. (**F**): SOD activity. (**G**): Proline content. (**H**): Soluble sugar content. (**I**): Chlorophyll content. Note: *, **, and *** denoting significant differences at *p* < 0.05, 0.01, and 0.001 levels, respectively.

To investigate how plants respond to drought stress, four varieties of *Arabidopsis* plants (wild type WT, *SpBGLU25* overexpression, mutant *atbglu25*, and mutant complementation *SpBGLU25-atbglu25*) were exposed to 20% PEG stress for 12 h, 24 h, and 7 days. It was found that all *Arabidopsis* plants experienced varying levels of damage due to drought stress. Prior to treatment, all four varieties appeared healthy; however, after 12 h, all except the *SpBGLU25* overexpression plants exhibited slight yellowing. After 24 h, the leaves of the three non-transgenic displayed significant yellowing and wilting, with the *atbglu25* knockout plants suffering the most severe damage, while the *SpBGLU25* overexpression plants showed the least. After 7 days of drought treatment, the wild type, *atbglu25* knockout, and *SpBGLU25-atbglu25* complementation plants were nearly completely dried out and dead, whereas some *SpBGLU25* overexpression plants survived (Figure 4A).

To further assess the drought tolerance of the *SpBGLU25* transgenic *Arabidopsis*, this study evaluated various physiological indicators in the four types of plants: wild type (WT), *SpBGLU25* overexpression, *atbglu25* knockout, and *SpBGLU25-atbglu25* complementation. Measurements were taken under normal growth conditions and 24 h after the 20% PEG, focusing on indicators such as relative water content (RLWC), malondialdehyde (MDA), peroxidase (POD), superoxide dismutase (SOD), proline (Pro), soluble sugars, soluble proteins, and chlorophyll.

Under normal conditions, there were no significant differences in physiological indicators among the four *Arabidopsis* types. However, after the 20% PEG drought stress treatment, notable changes were observed. The *SpBGLU25* transgenic overexpression type had lower malondialdehyde levels compared to the wild type, while all other indicators were significantly elevated. Specifically, RLWC was 6.5413% higher than that of the wild type; peroxidase activity increased by 2044.8 U/g; superoxide dismutase activity rose by 29.509 U/g; soluble protein content was up by 17.198 μg/mL; proline content increased by 8.649 μg/mL; and chlorophyll content grew by 0.0443 mg/g; and soluble sugar content rose by 4.3909 mg/g (Figure 4B–I).

From these experimental results, it can be concluded that the *SpBGLU25* gene significantly boosts the drought tolerance of *Arabidopsis* by enhancing antioxidant defense and osmotic regulation, enabling transgenic plants to maintain a healthier physiological state under drought conditions.

### 2.4. SpBGLU25 Enhances Drought Resistance in Arabidopsis by Activating Phenylpropanoid Metabolism and ABA Signaling Pathways

**Figure 5:**
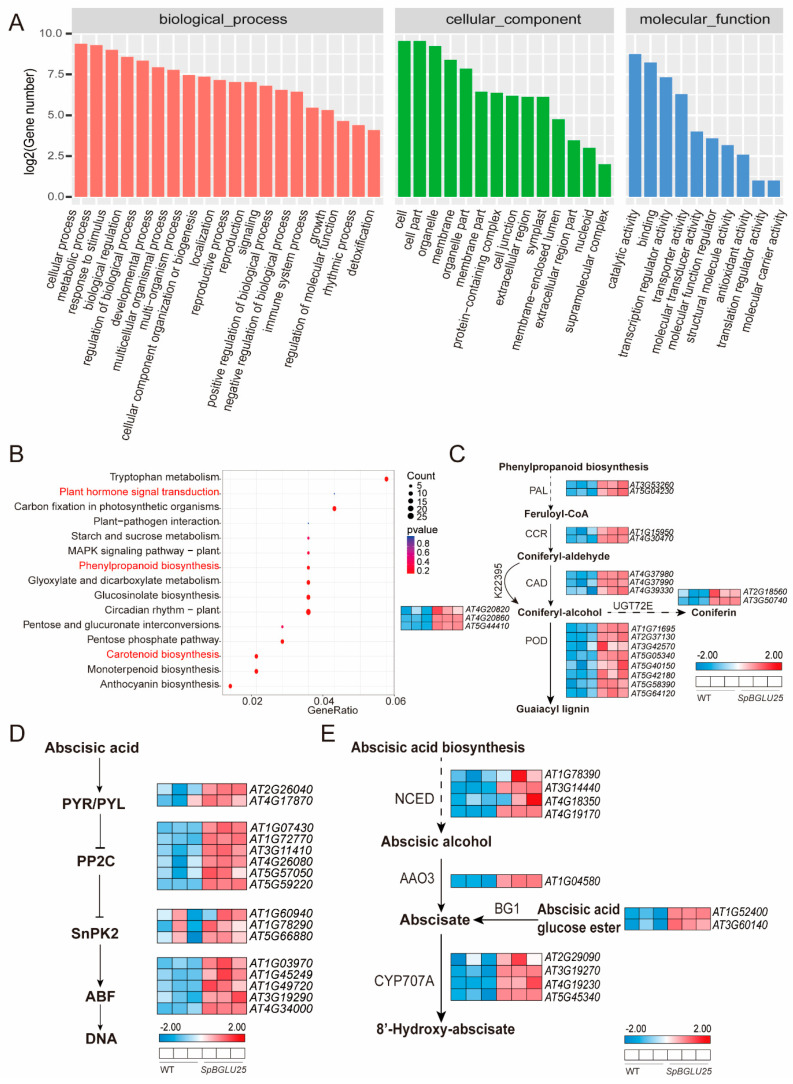
Transcriptomic analysis of differentially upregulated genes in *SpBGLU25*-overexpressing vs. wild-type (WT) *Arabidopsis*. (**A**): GO analysis of upregulated differentially expressed genes (UDEGs) in *SpBGLU25* vs. WT *Arabidopsis*. (**B**): KEGG enrichment analysis of UDEGs in *SpBGLU25* vs. WT *Arabidopsis*. (**C**): Expression levels of key phenylpropanoid biosynthesis pathway genes in *SpBGLU25* vs. WT. (**D**): Expression levels of ABA signaling pathway genes in *SpBGLU25* vs. WT. (**E**): Expression levels of ABA biosynthesis pathway genes in *SpBGLU25* vs. WT.

To further investigate the function of the *SpBGLU25* gene, we conducted transcriptome sequencing of both *SpBGLU25*-overexpressing and wild-type (WT) *Arabidopsis* plants. This was followed by Gene Ontology (GO) and Kyoto Encyclopedia of Genes and Genomes (KEGG) analyses of the upregulated differentially expressed genes (UDEGs) in the *SpBGLU25*_vs_WT comparison. The GO analysis revealed that the enriched terms primarily encompassed categories such as cellular processes, responses to stimuli, biological regulation, developmental processes, and metabolic activities. This suggests that *SpBGLU25* may influence the growth, development, and stress responses of *Arabidopsis* by modulating various cellular functions and biological processes (Figure 5A). Furthermore, the KEGG annotation analysis identified numerous genes involved in metabolic, genetic information processing, and signal transduction pathways (Figure 5B). A closer examination of the enrichment results indicated that the differentially expressed genes were predominantly concentrated in pathways related to tryptophan metabolism, plant hormone signal transduction, starch and sucrose metabolism, and phenylpropanoid biosynthesis. This implies that the *SpBGLU25* gene may enhance *Arabidopsis*’s drought resistance through various metabolic pathways. To verify the accuracy of the experimental findings from the transcriptome analysis, we focused on the phenylpropanoid metabolic pathway and conducted quantitative reverse transcription polymerase chain reaction (qRT-PCR) validation on the genes that were upregulated in the abscisic acid pathway. The results demonstrated a strong correlation with the transcriptome analysis (Figure S1).

An analysis of the metabolic pathways of the upregulated genes showed that 16 genes were significantly enriched in the phenylpropanoid metabolic pathway, which consists of two main branches: the lignin pathway and the flavonoid pathway. Lignin is a key component of the secondary cell wall in plant cells and is essential for plant growth, development, and stress resistance. Plants can fortify their cell walls by adjusting their components to withstand stressful conditions. Transcriptome analysis identified several key enzyme genes associated with lignin biosynthesis, including *phenylalanine ammonia lyase* (*PAL*), *cinnamoyl-CoA reductase* (*CCR*), *cinnamyl alcohol dehydrogenase* (*CAD*), *peroxidase superfamily protein* (*POD*), and *UDP-glucosyl transferase* (*UGT72E*), all of which were significantly upregulated (Figure 5C). Among these, *CCR* and *CAD* facilitate the production of coniferyl alcohol, *UGT72E* aids in the synthesis of coniferin, and *POD* promotes the formation of guaiacyl lignin. This indicates that the *SpBGLU25* transgene can enhance lignin production, leading to the lignification of *Arabidopsis* cell walls, which helps reduce water loss and mitigate drought stress. Additionally, in the plant hormone signaling pathway, the primary pathway involved is the abscisic acid (ABA) pathway, with upregulated genes including ABA receptors (*PYR/PYL*), protein phosphatases (*PP2C*), *sucrose non-fermenting protein kinase* (*SnPK2*), and *ABA-responsive element binding factors* (*ABF*) (Figure 5D). Carotenoids serve as antioxidants that help minimize oxidative damage to plants under various stress conditions. The abscisic acid generated during carotenoid metabolism is a crucial hormonal substance in plants, playing a significant role in growth, development, and stress resistance. Transcriptomic analysis revealed significant upregulation of key ABA biosynthesis pathway genes in *SpBGLU25*-overexpressing *Arabidopsis* plants, including *nine 9-cis-epoxycarotenoid dioxygenases* (*NCEDs*), *abscisic aldehyde oxidase 3* (*AAO3*), *cytochrome P450 707A* (*CYP707A*), and *β-glucosidase 1* (*BG1*) (Figure 5E). These findings strongly suggest that *SpBGLU25* likely enhances drought tolerance in *Arabidopsis* by promoting ABA biosynthesis.

### 2.5. Detection of Germination Rate and Root Length of Four Arabidopsis Types Seeds Under ABA Treatment

**Figure 6:**
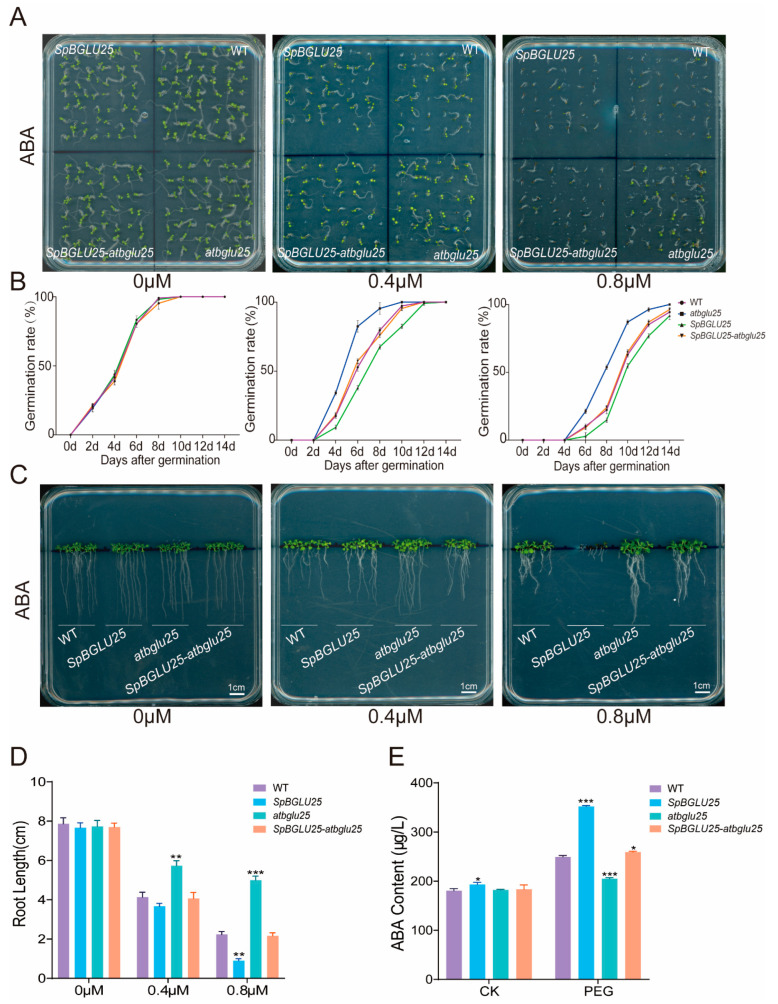
Effects of ABA treatment on seed germination and root development in *SpBGLU25*, WT, *atbglu25*, and *SpBGLU25-atbglu25 Arabidopsis*. (**A**): Phenotypic images of germination rates of four *Arabidopsis* types under different concentrations of ABA treatment. (**B**): Statistical chart of germination rates of four *Arabidopsis* types under different concentrations of ABA treatment. (**C**): Phenotypic images of root lengths of four *Arabidopsis* types under different concentrations of ABA treatment. (**D**): Statistical chart of root lengths of four *Arabidopsis* types under different concentrations of ABA treatment. (**E**): Detection of ABA content in four *Arabidopsis* types under PEG treatment. Note: *, **, and *** denoting significant differences at *p* < 0.05, 0.01, and 0.001 levels, respectively.

To investigate the relationship between the *SpBGLU25* gene and abscisic acid (ABA), experiments were conducted by incorporating various ABA concentrations (0 μM, 0.4 μM, 0.8 μM) into 1/2 MS medium. The findings revealed that as ABA concentration increased, the germination rate of plants overexpressing *SpBGLU25* was slower compared to that of the *atbglu25* knockout mutant *Arabidopsis* seeds (Figure 6A). The germination rate for *SpBGLU25* overexpressing plants reached nearly 100% by the 12th and 14th days, while the *atbglu25* knockout mutant seeds achieved similar rates by the 8th and 12th days, respectively (Figure 6B). Furthermore, higher ABA concentrations inhibited root lengths across all four *Arabidopsis* types, with the *SpBGLU25* transgenic type experiencing more significant inhibition than both the wild type and the *atbglu25* knockout mutant (Figure 6C,D). This observation supports the hypothesis that β-glucosidase can convert ABA-GE into biologically active free ABA. The application of exogenous ABA leads to a saturation of intracellular ABA levels in *SpBGLU25* overexpressing plants, which significantly hampers seed germination and reduces root length in *Arabidopsis*. ABA is a crucial hormone in plants’ responses to drought stress. To further examine the impact of the *SpBGLU25* gene from *S. pennata* on the drought resistance of *Arabidopsis thaliana*, we utilized four types of *Arabidopsis*: wild type (WT), *SpBGLU25* overexpressing type, *atbglu25* knockout type, and *SpBGLU25—atbglu25* complementation type. We assessed the ABA content under drought stress. The results indicated that the ABA content in the *SpBGLU25* overexpressing type was slightly higher than in WT under normal conditions, and under dehydration conditions, the ABA content increased by 102.605 µg/L (Figure 6E), demonstrating drought tolerance. These results suggest that the *SpBGLU25* gene likely enhances drought tolerance in *Arabidopsis* by promoting the release of bioactive ABA.

## 3. Discussion

Climate change-induced abiotic stress results in reduced crop yields and production [27]. One significant abiotic stress is soil drought, which hampers the growth, development, and yield of crops globally [28,29]. Current climate trends show increasing temperatures and declining annual rainfall, suggesting that future crops will encounter more severe soil droughts [30]. To adapt to arid conditions, plants have evolved various morphological, physiological, biochemical, and molecular strategies, all regulated by multiple genes [31,32,33]. Identifying key genes that improve drought resistance is therefore crucial.

In the intricate processes of plant growth, development, and response to abiotic stress, β-glucosidase is vital. This study focuses on the sand-fixing plant *S. pennata*, from which the β-glucosidase gene *SpBGLU25* was successfully cloned. This not only adds to the plant’s genetic resources but also sets the stage for deeper exploration of this gene’s unique functions in desert plants. Analyzing the gene’s characteristics reveals that *SpBGLU25* encodes a hydrophilic protein consisting of 193 amino acids, located in the cell membrane. This localization may be significant for substance exchange and signal transduction, thereby aiding in drought stress response. qRT-PCR analysis shows a strong correlation between its expression and the drought resistance of *S. pennata*, while overexpression studies in transgenic *Arabidopsis* demonstrate a marked increase in drought resistance. Various physiological indicators further support these findings, aligning with previous research on β-glucosidase’s role in plant stress resistance and underscoring the enzyme’s importance in drought response.

GO and KEGG analyses identified the biological processes and metabolic pathways associated with the *SpBGLU25* gene. The differentially expressed genes were enriched in categories such as “cellular process,” “cell,” and “catalytic activity,” as well as in key metabolic pathways like phenylpropanoid biosynthesis, plant circadian rhythm, carotenoid biosynthesis, and glycolic and dicarboxylic acid metabolism. This indicates that the *SpBGLU25* gene enhances plant drought resistance through coordinated actions across multiple pathways. Notably, the phenylpropanoid biosynthesis pathway is linked to lignin production, which may strengthen plant cell walls and improve stress resilience, while the carotenoid biosynthesis pathway is connected to ABA synthesis, potentially enhancing drought resistance through hormonal regulation.

### 3.1. The Transgenic Arabidopsis SpBGLU25 Adapts to Drought Stress by Increasing Phenolic Compounds and Cell Wall Strength

Phenolic compounds are essential antioxidants that accumulate in plants under abiotic stress, aiding their adaptation to challenging environments and playing a significant role in stress resistance [27,34]. The enzyme phenylalanine ammonia-lyase (PAL) is one of the first in the phenylpropanoid biosynthetic pathway and is crucial for the production of various secondary metabolites, including flavonoids, lignin, and phenolic compounds [35]. For example, in *Salvia miltiorrhiza*, drought stress results in a significant increase in *PAL* gene expression and a rise in polyphenolic compounds, which help combat oxidative stress [36]. This research indicates that the overexpression of the *PAL3* gene in *Arabidopsis* is notably elevated, suggesting that it may facilitate the accumulation of phenolic compounds by modulating the expression of essential enzyme genes in the phenylpropanoid metabolic pathway to manage excess reactive oxygen species (ROS) and cope with drought stress.

Lignin, a key component of plant cell walls, exhibits a positive correlation with drought stress, enhancing water transport and minimizing water loss [37]. The enzyme CAD (cinnamate-4-hydroxylase) is crucial for lignin synthesis and plays a significant role in the lignin production pathway [38]. Drought conditions typically lead to increased lignin deposition, which is associated with heightened *CAD* activity and gene expression [39]. This study found that *CAD* expression levels were significantly elevated, suggesting that it may assist plants in adapting to drought stress by promoting lignin synthesis and strengthening cell walls. In *Arabidopsis*, members of the uridine diphosphate-dependent glycosyltransferase UGT72E family have been identified as glycosylating lignin monomers [40]. The degree of glycosylation of lignin precursors is a critical mechanism for regulating the transport of these monomers, which is essential for lignin synthesis [41]. Currently, glycosyltransferases UGT72E1-3 in *Arabidopsis* have been shown to catalyze reactions involving phenolic alcohol derivatives, particularly sinapyl alcohol and coniferyl alcohol [42]. The formation of glycosides aids in the transmembrane transport of lignin precursors, thus influencing lignin synthesis.

In this study, the expression of the *UGT72E1* gene in *SpBGLU25* transgenic *Arabidopsis* was significantly elevated, indicating its role in regulating lignin synthesis to enhance the plant’s ability to withstand drought stress. Peroxidases play a crucial role in preventing the accumulation of H_2_O_2_ in cells and mitigating its toxic effects [43]. For instance, the expression of *PRX52* in cabbage is significantly increased, which contributes to the removal of reactive oxygen species (ROS) and enhances the plant’s response to abiotic stress [44]. The *PER64* gene in arbuscular mycorrhiza is crucial for salt tolerance [45]. This study observed a notable increase in the expression levels of peroxidase genes within the phenylpropanoid metabolic pathway, suggesting a connection to ROS removal and, consequently, improved drought resistance.

Drought stress adversely affects plants by causing cell dehydration, accumulating reactive oxygen species (ROS), and damaging cellular membranes, which can ultimately lead to plant death. The *atbglu25* mutant, which has a defect in lignin production, struggles to withstand this damage, resulting in higher mortality rates. In contrast, lines that overexpress *SpBGLU25* exhibit reduced damage when subjected to drought stress. In conclusion, the *SpBGLU25* transgenic *Arabidopsis* enhances the accumulation of phenolic compounds, lignin, and other substances by regulating the expression of key genes in the phenylpropanoid metabolic pathway. This process helps mitigate ROS damage and strengthens cell walls through lignin synthesis, enabling the plant to better adapt to drought stress.

### 3.2. Transgenic Arabidopsis SpBGLU25 Adapts to Drought Stress Through Plant Hormone Signaling

Plant hormone signaling plays a vital role in how plants respond to stress, with endogenous hormones being essential for managing both biotic and abiotic challenges [46]. Various endogenous hormones have distinct functions during stress responses. Abscisic acid (ABA) is a major regulatory component involved in numerous physiological and developmental processes [29]. It can be synthesized through a complex de novo pathway or produced via the hydrolysis of inactive ABA-glucosyl ester (ABA-GE) [18]. Research conducted by Lee et al. identified the enzyme *β-glucosidase* gene (*BGLU18*) in *Arabidopsis*, which responds to drought stress by hydrolyzing ABA-GE to release free ABA from the endoplasmic reticulum [22]. ABA is generated or accumulated in the guard cells surrounding the stomata, resulting in stomatal closure that reduces water loss and enhances drought resistance [47]. Additionally, the enzyme nine-cis-epoxycarotenoid dioxygenase (NCED) is crucial for producing ABA by cleaving carotenoids [48]. The study demonstrated that the expression levels of key genes *BG1*, *NCED*, and *AOA3* in the carotenoid biosynthesis pathway of *SpBGLU25* transgenic *Arabidopsis* were significantly elevated, suggesting a strong link between *SpBGLU25* and ABA, which responds to drought stress by increasing ABA production.

In conclusion, *SpBGLU25* transgenic *Arabidopsis* significantly enhances the expression of key enzymes and transcription factors involved in hormone synthesis, thereby modulating plant hormone levels to mitigate drought stress. This study suggests that *SpBGLU25* likely influences the plant’s drought response by regulating ABA metabolism. It promotes the hydrolysis of ABA-GE, leading to higher free ABA levels and activation of the ABA signaling pathway. This activation leads to stomatal closure, the accumulation of osmotic adjustment substances, and improved drought resistance. Furthermore, components of the ABA signaling pathway may also affect the expression of the *SpBGLU25* gene, establishing a complex feedback regulatory network.

Overall, the results of this research offer new insights and genetic resources for enhancing plant stress resistance through genetic engineering, with important theoretical and practical implications.

## 4. Materials and Methods

### 4.1. Materials

#### 4.1.1. Plant Materials

In June, *S. pennata* seeds were gathered using bagging techniques in the desert close to the Mosowan Reservoir in Shihezi City, located in the Xinjiang Uygur Autonomous Region. The seeds were then dehulled and treated with gibberellin before being sown in sandy soil to grow full plants for experimental purposes.

#### 4.1.2. Experimental Reagents

The total RNA extraction kit, cDNA first strand reverse transcription kit, 2×Taq PCR Master Mix II, DNA gel recovery kit, and plasmid extraction kit utilized in this study were acquired from TIANGEN (TIANGEN Biotech (Beijing) Co., Ltd., Beijing, China). The *pMD-19 T* cloning vector and real-time PCR reagents were sourced from TaKaRa Bio, along with enzymes like Kpn I and Xba I. Chemical reagents such as penicillin ampicillin, kanamycin, gentamicin, MES, acetosyringone, MgCl_2_, and components for culture media were all domestic analytical grade products from Shanghai Shenggong Biological Engineering Company (Shanghai Shenggong Bioengineering Company, Shanghai, China). The Agrobacterium strain GV3101 and the subcellular localization vector *pCAMBIA 1300* were stored at the Key Laboratory of Agricultural Biotechnology at Shihezi University in Xinjiang, while the competent E. coli strain DH 5α was obtained from Beijing Quanshijin Bio (Beijing Quanshi Jin Biological Company, Beijing, China). The synthesis of PCR primers and DNA sequencing was carried out by Xinjiang Youkang Biotechnology Co., Ltd (Xinjiang Youkang Biotechnology Co., Ltd., Xinjiang, China). and Shanghai Shenggong Biological Engineering Co., Ltd (Shanghai Shenggong Bioengineering Company, Shanghai, China). Kits for MDA, SOD, POD, chlorophyll, soluble sugars, and Pro were purchased from Solarbio Life Sciences (Beijing Solarbio Science & Technology Co., Ltd., Beijing, China), while kits for ABA and soluble proteins were sourced from Jingmei Co., Ltd (Jiangsu Jingmei Biotechnology Co., Ltd., Jiangsu, China).

### 4.2. Methods

#### 4.2.1. Planting of *S. pennata* Seeds

Once the gathered *S. pennata* seeds have been dried and the seed coat has been removed, immerse them in gibberellin for 24 h. Subsequently, plant the seeds 1 cm deep in sandy soil. After 45 days, gather samples that have been treated with a 500 mmol/L mannitol solution for durations of 0 h, 3 h, 6 h, 12 h, and 24 h for additional analysis.

#### 4.2.2. RNA Extraction and cDNA Synthesis of *S. pennata*

Adhere to the guidelines provided by the TIANGEN plant total RNA extraction kit for the extraction process. Once the extraction is complete, verify the integrity of the RNA with 1.1% agarose gel electrophoresis and determine the RNA concentration. Next, use a reverse transcription kit to synthesize cDNA. Store the cDNA at –20 °C.

#### 4.2.3. Cloning of the *SpBGLU25* Gene

*SpBGLU25* was identified from transcriptome data based on earlier laboratory studies, and its complete coding sequence (CDS) was acquired. Primers *SpBGLU25*-F and *SpBGLU25*-R were created, along with homologous arm primers *SpBGLU25*-tong-F and *SpBGLU25*-tong-R that included KpnI and XbaI restriction sites. Using cDNA from *S. pennata* as a template, PCR amplification was carried out with the following reaction mixture: 2 µL of cDNA (50 ng·µL^−1^), 25 µL of 2×Taq PCR Master Mix, 2 µL of primer *SpBGLU25*-F, 2 µL of primer *SpBGLU25*-R, and 19 µL of ddH_2_O, totaling 50 µL. The amplification protocol consisted of an initial step at 95 °C for 5 min, followed by 35 cycles of 95 °C for 30 s, 51 °C for 30 s, and 72 °C for 1 min, concluding with a final extension at 72 °C for 5 min, and then stored at 4 °C. A 1.2% agarose gel was utilized for electrophoresis to visualize and isolate the target band, which was subsequently sent to Xinjiang Youkang Biotechnology Co., Ltd. (Xinjiang Youkang Biotechnology Co., Ltd., Xinjiang, China) for sequencing.

#### 4.2.4. Gene Sequence Analysis

The sequence alignment analysis was carried out using DNAMAN software (Version 9). The conserved domain analysis for the feather needle wheat *SpBGLU25* was conducted with the online tool ConservedDomains available on the NCBI website. The physicochemical properties of the protein encoded by *SpBGLU25* were analyzed and calculated using the PortParam online tool from the ExPASy server (https://web.expasy.org/protparam/, accessed on 10 October 2024) [49]. Hydrophobicity analysis of the protein was performed using ProtScale (http://web.expasy.org/protscale/, accessed on 10 October 2024 [49]. The prediction of the protein’s secondary structure was performed using the phyre2 online tool (http://www.sbg.bio.ic.ac.uk/phyre2/, accessed on 10 October 2024) [50], while the tertiary structure prediction was carried out using alphafold (https://alphafold.ebi.ac.uk/) [50]. The subcellular localization of the protein was analyzed using the Plant-mPLoc online tool (http://www.csbio.sjtu.edu.cn/bioinf/plant-multi/, accessed on 10 October 2024) [51]. Evolutionary analysis of the protein was performed using MEGA (Version 11) [52], motif analysis was conducted using the MEME online tool [53], and the visualization of evolution, conserved domains, and motifs was performed with TBtools [54].

#### 4.2.5. Construction of Plant Expression Vectors

The properly aligned gel recovery products were introduced into DH 5α competent cells using a homologous recombination kit, and then plated on LB solid medium with kanamycin. After a 12 h incubation at 37 °C, positive monoclonal colonies were chosen for colony PCR verification and sent to Xinjiang Youkang Biotechnology Co., Ltd. for sequencing. The sequencing results were compared to the original sequence using SnapGene, and the correctly aligned bacterial culture was used to extract plasmids following the plasmid extraction kit’s instructions. These plasmids were then transformed into Agrobacterium GV3101 via a freeze–thaw method and plated on LB solid medium containing three antibiotics (Gen 50 μg·mL^−1^, Kan 50 μg·mL^−1^, Rif 50 μg·mL^−1^), followed by incubation at 28 °C for 36–48 h. Single colonies were selected for colony PCR verification, and the correctly verified Agrobacterium culture was preserved.

#### 4.2.6. Subcellular Localization

The recombinant plasmid that was successfully created was cultured in LB liquid medium with rifampicin, gentamicin, and kanamycin at a speed of 200 r/min and a temperature of 28 °C for 24 h. Afterward, the cells were harvested by centrifugation at 5000 r/min for 10 min to obtain a cell pellet. This pellet was then resuspended in MS liquid medium containing 100 mmol·L^−1^ MES, 10 mmol·L^−1^ MgCl_2_, and 100 μmol·L^−1^ AS to achieve an OD600 of 1.

Fresh onions were peeled to remove the outer three layers of scales, and the inner scales were cut into small 1 cm^2^ pieces. These pieces were soaked in the prepared MS liquid suspension and shaken at 200 r/min and 28 °C for 45 min. Under sterile conditions, the onion scale pieces were then carefully placed flat on MS solid medium and incubated in the dark at room temperature for 2 days. During the observation phase, the inner epidermis of the onion was gently torn to prepare slides, which were then examined and photographed using a laser confocal microscope. Subcellular localization prediction was conducted using the online tools WOLF PSORT and Cell-PLoc (accessed on 10 October 2024), with results indicating that the protein is mainly localized in the cell membrane, suggesting that this gene operates within the cell membrane.

#### 4.2.7. Gene Expression Characterization Analysis

*S. pennata* samples were gathered from the roots, nodes, leaves, and seeds, including roots that underwent drought stress treatment at intervals of 0 h, 3 h, 6 h, 12 h, and 24 h. RNA was extracted from these samples, and cDNA was produced through reverse transcription. The GAPDH gene was chosen as the reference gene. Primers for *SpBGLU25* were designed using the NCBI online tool (see Table 1). qRT-PCR was conducted using the reverse-transcribed cDNA as a template, following the guidelines of the fluorescent quantitative premix reagent (SYBR Green) (TIANGEN Biotech (Beijing) Co., Ltd., Beijing, China). The reaction mixture consisted of 10 μL total: 5 μL of 2× SuperReal PreMix Plus (TIANGEN Biotech (Beijing) Co., Ltd., Beijing, China), 0.5 μL of forward primer, 0.5 μL of reverse primer, 2.5 μL of cDNA template, and 1.5 μL of RNase-free ddH_2_O. The amplification protocol included an initial pre-denaturation at 95 °C for 15 min, followed by 95 °C for 10 s for denaturation, 52 °C for 20 s for annealing, and 72 °C for 20 s for extension, repeated for 38 cycles, with three replicates for each sample. The relative expression level of the target gene was determined using the 2^–ΔΔCt^ method.

#### 4.2.8. Obtaining and Identifying *SpBGLU25* Gene Overexpression in *Arabidopsis*

Utilize the floral dip technique with Agrobacterium tumefaciens to introduce the successfully created recombinant plasmid into *Arabidopsis thaliana*. Select for positive seedlings on 1/2 MS medium supplemented with 4% hygromycin, gather seeds once they are fully developed, and persist in screening on hygromycin-enriched medium until you achieve homozygous T3 generation positive seedlings that overexpress *SpBGLU25*.

#### 4.2.9. The Effect of Drought Stress on the Phenotype of Arabidopsis Overexpressing the *SpBGLU25* Gene

A 20% PEG solution can mimic the water potential found in moderately dry soil, leading to observable changes in plants, like yellowing leaves and wilting, as well as notable physiological reactions, including the buildup of reactive oxygen species (ROS) and the peroxidation of membrane lipids. This study aims to explore how drought stress in soil affects the phenotype of *Arabidopsis* plants that overexpress the *SpBGLU25* gene. Choose four varieties of *Arabidopsis* that have the same growth condition: wild type, transgenic type, knockout type, and complemented type. Plant them in a mixture of nutrient soil, vermiculite, and perlite. After twenty days, apply a drought treatment by irrigating with 20% PEG, and then observe the phenotypes and collect samples after 12 and 24 h of treatment. Following the stress treatment, gather plant samples at 0 and 24 h to analyze the levels of RLWC, MDA, ABA, Pro, soluble sugars, soluble proteins, chlorophyll, and the activities of SOD, POD, and other relevant enzymes.

#### 4.2.10. Determination of Germination Rate and Root Length of *SpBGLU25* Gene Overexpressing *Arabidopsis* Seeds

To investigate the seed germination of transgenic *SpBGLU25* lines under varying levels of drought stress in sterile conditions, seeds from wild-type, transgenic lines, knockout, and complementation *Arabidopsis* were sown in 1/2 MS medium with different concentrations of mannitol (0, 150, 300 mM). Additionally, to explore the relationship between the *SpBGLU25* gene and ABA, the four types of *Arabidopsis* seeds were planted in 1/2 MS medium with varying ABA concentrations (0, 0.4, 0.8 μM) and grown under normal conditions, with daily monitoring of germination rates. To measure root length, seeds were planted in the medium and allowed to grow vertically for two weeks before assessing root length. Each sample included three biological replicates.

#### 4.2.11. Transcriptome Analysis

Following the disinfection and washing of the WT and *SpBGLU25 Arabidopsis* seeds, they were sown on 1/2 MS solid medium. After a period of ten days, the seedlings were moved to nutrient soil and allowed to grow for an additional 18–20 days to harvest the *Arabidopsis* plants, ensuring three biological replicates for each variant. The samples were subsequently shipped to Wuhan Aijibai Biological Company for transcriptome sequencing using dry ice for transportation.

#### 4.2.12. Statistical Analysis

All calculations and analyses were conducted using Excel and GraphPad Prism 9.5.0 software, following these specific steps: Each physiological and biochemical index assessed under normal and drought stress conditions was repeated three times. The data were organized and statistically analyzed in Excel, while significance testing was performed using two-way ANOVA in GraphPad Prism 9.5.0, which also facilitated the creation of graphs.

## 5. Conclusions

In this research, the *β-glucosidase* gene *SpBGLU25* was isolated from the sand-stabilizing plant, *S. pennata*. Detailed analysis revealed that the gene product is situated in the cell membrane. Comprehensive experimental studies demonstrated that the *SpBGLU25* gene can effectively respond to drought conditions, significantly improving the plant’s drought tolerance. Initial findings indicate that this gene may break down ABA-GE (abscisic acid-glucosyl ester) to generate biologically active ABA (abscisic acid) and primarily produce lignin through the phenylpropanoid metabolic pathway, thereby enhancing the plant’s resilience. Furthermore, the *SpBGLU25* gene collaborates with other metabolic pathways to finely tune the plant’s response to drought stress. The results of this study offer new insights into the molecular mechanisms underlying plant drought resistance and are anticipated to provide essential genetic resources and theoretical foundations for the genetic engineering of plant stress tolerance.

## Figures and Tables

**Table 1:** Primers used in this study.

Primer Name	Primer Sequence (from 5′ to 3′)	Primer Function
*SpBGLU25*-F	ATGGAAGTACTTTGTGAAACC	Gene cloning
*SpBGLU25*-R	CCTGCGGTCATTGTCAT	
*SpBGLU25*-tong-F	atttggagaggacagggtaccATGAAGGACATTGGCATGGATG	Gene vector construction
*SpBGLU25*-tong-R	ggtactagtgtcgactctagaAAGTCCATTGCTCGTGATGCTG	
q-GAPDH-F	AGTCCGTCGCCATCGTCA	The reference gene
q-GAPDH-R	CGTGCCCATGCCTTCTGT	
q-*SpBGL25*-F	AGCTCATGCTGGTGCTTTTC	Gene expression analysis
q-*SpBGL25*-R	GTCCATTGCTCGTGATGCTG	

## Data Availability

All data are presented in the article and the Supplementary Materials.

## Supplementary Materials

The following supporting information can be downloaded at: https://www.mdpi.com/article/10.3390/ijms26146663/s1.
