# Supplementary material for: Overexpression of the β-Glucosidase Gene SpBGLU25 from the Desert Pioneer Plant Stipagrostis pennata Enhances the Drought Tolerance in Arabidopsis
Source: Int J Mol Sci. 2025 Jul 11;26(14):6663. doi: 10.3390/ijms26146663 (PMC12295429; doi:10.3390/ijms26146663)
Supplement: Supplementary file 1 [file ijms-26-06663-s001.zip › ijms-3739068-supplementary.pdf]

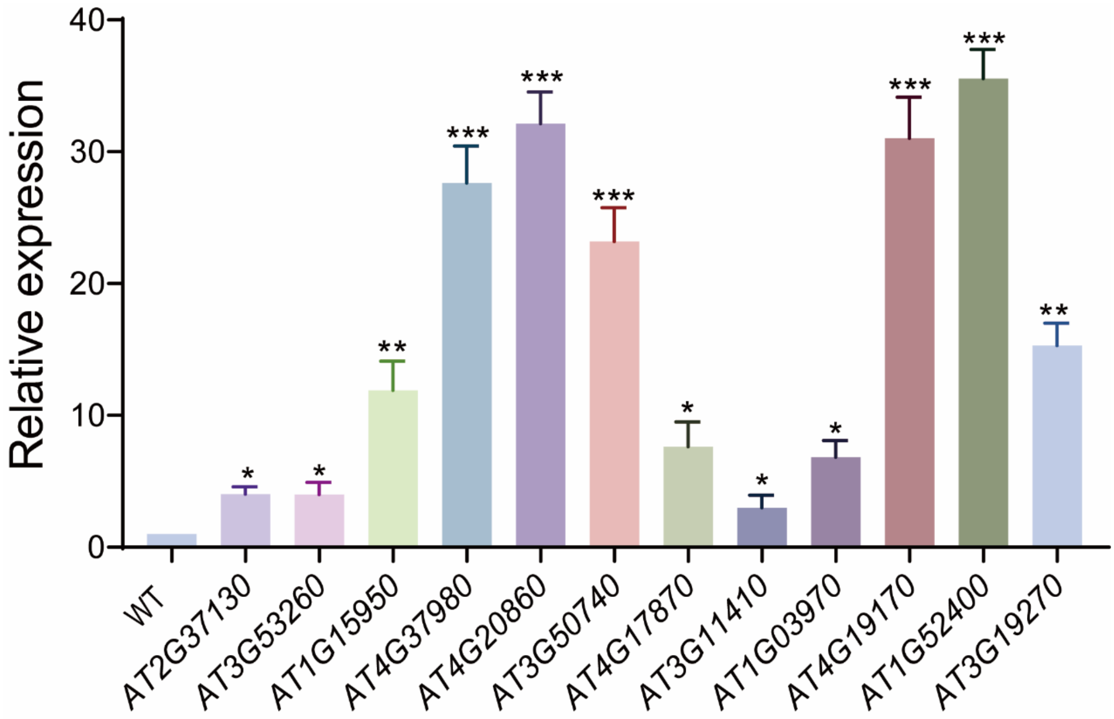

Figure S1. qRT-PCR validation of differentially expressed genes upregulated in the phenylpropanoid metabolic pathway, ABA signaling pathway, and ABA biosynthesis pathway.

Note: *, **, and *** denoting significant differences at $p < 0.05$, 0.01, and 0.001 levels, respectively.
